# Determining specific competencies for General Internal Medicine residents (PGY 4 and PGY 5). What are they and are programs currently teaching them? A survey of practicing Canadian General Internists

**DOI:** 10.1186/1756-0500-4-480

**Published:** 2011-11-03

**Authors:** Sharon E Card, Anne M PausJenssen, Rachel C Ottenbreit

**Affiliations:** 1 Division of General Internal Medicine, Department of Internal Medicine, University of Saskatchewan, 103 Hospital Drive, Saskatoon, Saskatchewan, S7N OW8, Canada; 2 Medical Student, University of Saskatchewan, Saskatoon, Saskatchewan, S7N 0W8, Canada

## Abstract

**Background:**

General Internal Medicine (GIM) has recently been approved as a subspecialty by the Royal College of Physicians and Surgeons of Canada. As such, there is a need to define areas of knowledge that a General Internist must learn in those two years of training. There is limited literature as to what competencies are needed in a GIM practice. Draft competencies for GIM (4^th ^and 5^th ^year residents in internal medicine) training were developed over eight years with input from many stakeholders. Practicing General Internists were surveyed and asked their perspective as to the level of importance of each of these competencies for GIM training. They were also asked if training gaps exist in current training programs. The survey was offered widely online in both English and French to gain perspectives from as many different contexts as possible.

**Results:**

157 General Internists, in practice on average for 15 years, responded from all of Canada's provinces and territories. Practice profiles were diverse (large urban centers to rural centers). The majority of the competencies surveyed were perceived as important to attain at least proficiency in. Perioperative care, risk reduction, and the management of common, emergent, and complex internal medicine problems were identified as key areas to focus training programs on, with respondents perceiving these should be mastered to an expert level. Training gaps were identified, most frequently in that of the manager role (example managing practice).

**Conclusions:**

This is the first study we are aware of to attempt to isolate the opinions of practicing Canadian General Internists as to the major competencies that should be mastered as a General Internist. We suggest that "generalism" in the context of GIM, does not mean a bit of knowledge about everything but that defined objectives for training in this 'newest' of Royal College subspecialties can be identified. This includes mastery of core areas such as perioperative care, risk reduction, and management of common, emergent and multiple internal medicine problems. The training gaps identified need to be addressed to ensure that General Internists continue to provide excellence in health care delivery.

## Background

"Value generalism" is one of the principles that have emerged from the Future of Medical Education in Canada (FMEC) Undergraduate report [1]. To do this the report suggests that MD education must focus on broadly based generalist content [1]. As the postgraduate report is underway generalism themes have also emerged [2] particularly as it relates to societal needs. It is also however widely recognized that the definition of "generalism" is not universally accepted [3]. Lay literature suggests that "generalists" should know something about everything, [4] but in the increasingly complex health care environment this is a very difficult task [5].

General Internal Medicine (GIM) has recently been approved as a subspecialty of Internal Medicine by the Royal College of Physicians and Surgeons of Canada. Our hypothesis has been that there are areas that a generalist in internal medicine must master, i.e. that a GIM specialist has a distinct knowledge base. In Canada, postgraduate training in Internal Medicine is four years in length. The first three years of training are considered "core" after which the resident enters a subspecialty or the fourth year of Internal Medicine training. Due to perceived needs by trainees and programs, these fourth and in many cases fifth years have become distinct "General Internal Medicine" Training programs. This has resulted in a need to establish the main areas of expertise for a General Internist, thus creating the objectives of training (core competencies) for the subspecialty of GIM. In this era of Competency Based Medical Education it is increasingly important to be clear as to the learning outcomes for each of our areas of training [6]. This is even more imperative now that GIM has been established as a distinct subspecialty.

Since a survey in 2006 [7] there has been no further definition in Canada in the published literature as to what the defining competencies of a GIM (post core 3 years) residency should be and whether current programs are meeting them. This study was designed to ascertain the opinion of practicing general internists as to their perceptions as to which competencies are most important for current General Internal Medicine graduates. We hypothesized that although the knowledge base of a General Internist is broad there is also a select group of competencies that should be emphasized in training. The study was also designed to assess the impression of current practicing General Internists as to whether they feel current residency training programs place an appropriate amount of emphasis on the potential objectives of training for General Internal Medicine.

## Methods

The competencies to be surveyed were developed over eight years of discussion amongst GIM (post core 4^th ^and 5^th ^year internal medicine) program directors, GIM Division Directors and the Canadian Society of Internal Medicine (CSIM). They had been refined into draft objectives in the CanMEDs format in anticipation of GIM becoming a recognized entity at the Royal College of Physicians and Surgeons of Canada. The draft objectives have been circulated and revised extensively over the last eight years by numerous individuals with expertise in both GIM and medical education. Practicing General Internists had had input if they were members of CSIM Council. The competencies are distinctly different and more robust than the Internal Medicine Objectives for a four year Internal Medicine specialist at that time [8]. Procedures chosen to be surveyed included those previously perceived to be important for General Internists in previous surveys [7].

This study asked practicing General Internists their perception as to whether the competencies in the objectives should be learned in a GIM training program and if so, to what level of mastery:

• Working Knowledge: Able to demonstrate core aspects of disorder. Would be able to manage for a short time (hours) then need assistance.

• Proficient: Able to demonstrate working knowledge enhanced by ability to teach, consult, assess, and manage referrals. Would manage these areas alone with minimal subspecialty input.

• Expert: Detailed and sophisticated understanding which leads to advanced teaching and consultation on complex referrals. Readily able to apply and demonstrate familiarity and apply the scientific literature. Subspecialty input not required for day to day care of the patient.

The level of mastery was adapted from the Royal College of Physicians and Surgeons documentation for other programs, namely that of psychiatry. Participants were also asked if they felt the current competency has sufficient emphasis in current training programs.

The survey was translated into French by the CSIM translator. The survey was entered online through the University of Saskatchewan survey system. Ethics approval was obtained from the University of Saskatchewan Behavioural Research Ethics Board. Consent was obtained from participants via an invitation letter at the start of the survey. Subsequent completion and return of the survey was then considered consent to participate. All information was aggregated anonymously, and individual responses remained confidential.

To reach a cross-section of general internists the survey was promoted in the headline of the CSIM website, appeared twice in the CSIM e-newsletter (May and June 2010), and was featured in the Information from the President of CSIM e-blast in June 2010. The survey was sent via e-blast to the CSIM membership and by special invitation to the Principal Investigator's known GIM colleagues and the CSIM council members.

Results are reported as percentage of respondents answering affirmatively to the questions. Agreement is indicated as greater than 50% of respondents agreeing on a certain category of knowledge.

## Results

Using the CSIM membership list as the sampling frame, the response rate was: 126/654 (19.3%) of non-resident members and 31/437 of resident members (7.1%). The membership numbers includes some CSIM members who are not General Internists, or not practicing clinicians, thus the estimate would be that the response rate of those who are practicing GIM is higher than this.

The demographics of the respondents indicate that we were able to reach a cross section of general internists in terms of practice profiles, geographic location and time in practice. Of the 157 respondents 92% answered in English and 8% in French. 19% of the respondents were residents (of these 77% were PGY 4 or 5). 81% of respondents indicated they practiced > 75% GIM. All provinces and the territories were represented. Average years in practice were 18 years (English) and 15 years (French). Practice profiles included large urban centers with population greater than 100,000 (69%); rural (7%); small urban (13%) and 11% greater than 100 km from a tertiary care center.

Table 1 summarizes all competencies (112) surveyed. The majority of respondents (> 50%) felt that knowledge in all the areas were needed to at least a proficient level. The only exception was cardiac transplantation indications/referral, which is the only competency for which > 50% indicated working knowledge only was needed. There were no competencies for which > 50% felt that they should not be included. There were 13 competencies for which there was not a clear majority opinion. For 11 of these, agreement was split between proficient and expert and if these two categories are combined the percentage of respondents agreeing is greater than 70%. For two competencies, aortic dissection (emergency recognition and management) and thorough examination of the urine, agreement was split between working knowledge (40 and 42% respectively) and proficient (41 and 35% respectively).

**Table 1:** Competencies > 50% of Respondents agreed should be mastered to expert or proficient.

Discipline	# of Items	Greater Than 50% Agreed Expert	Greater than 50% Agreed Proficient
Cardiac	16	4	9

Pharmacology/Toxicology	5	1	4

Endocrinology	5	3	2

Geriatrics	4	1	3

Hematology	4	1	3

Infectious Diseases	5	2	3

Critical Care	3	0	3

Oncology	2	0	2

Nephrology	6	1	4

Neurology	3	0	2

Palliative Care	2	0	2

Respirology	4	1	3

Rheumatology	1	0	1

Rheumatology	1	0	1

Multiple Comorbidities	2	2	0

Risk reduction	6	4	2

Undifferentiated	1	1	0

Perioperative Care	12	9	0

Pregnancy	7	0	5

Communication	3	1	1

Collaboration	6	3	1

Manager	6	2	4

Advocate	3	0	3

Scholar	2	1	1

Professional	3	1	1

**TOTAL**	**112**	**38**	**60**

Table 2 summarizes those areas that > 50% of general internists felt should be learned to an expert level. Perioperative care, risk reduction, common and emergency internal medicine diagnoses and multiple internal medicine diagnoses were a consistent theme.

**Table 2:** Competencies > 50% of respondents indicated should be learned at the expert level.

**RISK REDUCTION**
Risk Factors for Coronary Artery Disease, Hypertension, Obesity and Syndrome X, Lipid Disorders.
**PERIOPERATIVE MANAGEMENT OF...**
Coronary Artery Disease, Multiple Co morbidities, Steroids, Acute Illness, Medications, Diabetes, Thromboprophylaxis, Prophylactic Antibiotics, Pulmonary Risk.
**COMMON INTERNAL MEDICINE DIAGNOSES**
Lower Respiratory Tract Infections, Acute and Chronic Heart Failure, Thrombosis, Delirium, Syncope Management of Type 1 and 2 Diabetes Mellitus, Diastolic Dysfunction
**EMERGENCY INTERNAL MEDICINE DIAGNOSES**
Diabetic Ketoacidosis and Hyperosmolar NonKetotic State Management of Alcohol Withdrawal Syndromes, Acute Infectious Emergencies, Electrolyte Disturbances
**MULTIPLE DIAGNOSES IN ONE INDIVIDUAL**
Communication, Multiple Co morbidities, Complex Chronic Care, Coordinate multiple interventions
**CHARACTERISTICS**
Knowing one's own limits of competence Manage time to balance patient care and personal life Work with others to provide care Ongoing Learning Undifferentiated presentations Advocacy for patient in challenging situations Skills in intra professional teams

Many items were felt to be insufficiently emphasized in current general internal medicine training programs - see table 3. This table indicates the gaps felt to be apparent in current GIM training programs.

**Table 3:** Competencies that have perceived gaps in training according to > 50% of respondents.

Competency	% Respondents
**MANAGER**	

Manage practice	79%

Manage balance	71%

Participation in quality improvement projects	59%

Patient safety initiatives	58%

Cost appropriate care	57%

**MEDICAL EXPERT**	

Drug Interactions	55%

Pharmacology in the elderly	52%

Pain Control	51%

Emergency Cardiac Syndromes in Pregnancy	51%

Proficiency in Exercise Stress Testing	50%

Knowledge of risk benefit for classes of medications in pregnancy	50%

**HEALTH ADVOCATE**	

Knowledge of Provincial Driving Restrictions	65%

**PROFESSIONAL**	

Recognize and respond to others' unprofessional behaviours	52%

Few procedures (of 38 possible options) were felt to be quite or very important by over 50% of respondents (see table 4). In contrast 18 procedures (all of which had been cited in previous studies as potentially being done by General Internists) were indicated by less than 40% of respondents to be important for General Internists to learn (example thyroid fine needle aspirate, liver biopsy, renal biopsy). Ninety-three percent of respondents thought that ultrasound guided procedures should be taught in GIM training programs.

**Table 4:** Procedures that > 50% of respondents think are quite/very important

Procedure	**Percent Agreeing quite or very important**.
ACLS/CPR	91

Central Venous Catheter Insertion	93

Paracentesis	90

Endotracheal Intubation	89

Lumbar Puncture	89

Thoracentesis	87

Arterial access and blood gases	82

Ambulatory ECG	79

Cardioversion	77

Exercise Stress Testing	77

Hemodynamic Monitoring	76

Temporary Pacemaker Insertion	75

Mechanical Ventilation	75

Transthoracic Pacing	71

Arthrocentesis	70

Bone Marrow Aspiration and Biopsy	60

Chest Tube Insertion	58

Venipuncture	50

Many very thoughtful narrative comments were added. Themes included flexibility in training needed and the need to be able to obtain procedural competencies for smaller communities if that was to be the individual's future practice location. No other specific competencies were suggested.

## Discussion

Although the knowledge base required of a General Internist is broad it is possible to isolate the core knowledge that a General Internist must master. The vast majority of items surveyed were perceived to require at least proficiency by practicing General Internists. A further subset of competencies was perceived to require knowledge at an expert level: this includes perioperative care. As the number of individuals with chronic diseases who require surgery increases expertise in this area will be increasingly needed by the public. Risk reduction was another predominant theme - again, with the increase in chronic disease in the population expertise in this area needs to expand. It is not surprising that for a "generalist" mastery of common and emergent internal medicine diseases was supported as a need for learning. Generalists have traditionally been thought of as dealing with "the whole person" versus individual disease - this was again supported by the perception that mastery of multiple co morbidities in one individual is important. Obstetrical medicine (the care of pregnant women with internal medicine diseases) has been a contentious topic as to whether it is an area of needed knowledge for a General Internist. This study supports it as being an area that General Internists perceive they should be proficient in. Obstetrical Medicine has recently compiled a curriculum that is specific for obstetrical medicine knowledge base in GIM [9].

We suggest that with the deletion of only 3 competencies the draft objectives for GIM submitted to the Royal College of Physicians and Surgeons are considered appropriate by practicing general internists. For the majority of competencies agreement (as defined as > 50%) was established for the competency to either the level of proficient or expert. For all of the competencies with disagreement (i.e. no clear > 50%) but two the discrepancy was clearly between proficient and expert indicating still an area to be learned. We suggest that these competencies are within the domain of GIM and should form the objectives for the new subspecialty of GIM. We believe that our study supports the hypothesis that GIM is a distinct entity with a distinct knowledge base that must be mastered. This is contrary to the alternate thought that GIM is purely a compilation of small amounts of knowledge about many things. This is an important distinction for training programs suggesting that trainees should master the key knowledge base of GIM not simply rotate through many parts of Internal Medicine. The competencies included are distinct from those of an Internal Medicine specialist [8]. Attainment of knowledge in this area can be achieved in program specific ways but could include community GIM rotations, junior attending on GIM rotations, pre-assessment clinics, and GIM "fellows" clinics. GIM is distinctly different throughout the world and there is little previous literature which examines the perceived areas of knowledge that a Canadian General Internist should attain [10].

There is previous literature suggesting there is a gap in training in several of the areas pointed out in this survey as important, including perioperative care, by those who had recently trained in Canadian GIM training programs (at that time defined as 4^th ^year IM programs) [7]. This is the first study however to attempt to isolate the opinions of practicing Canadian General Internists as to what they feel the major competencies are that should be mastered as a General Internist. This study continues to point out perceived gaps in training by practicing General Internists. The manager role remains to be of significant concern to many, as it was in the 2006 study, suggesting that training programs need to develop training in this area. We suggest that this study supports the previous proposals by the CSIM and others that GIM specialists have detailed knowledge and skills sets that cross traditional boundaries of subspecialty medicine [11,12]. There were many procedures that have previously been proposed to be part of GIM training [7], however, in this study the procedures that the majority felt were quite or very important were those already listed in the Internal Medicine objectives plus life saving and emergent procedures (temporary pacemaker insertion) or those utilized frequently in risk reduction/common internal medicine diagnoses (exercise stress testing). This suggests that procedural training for all trainees in GIM should include these procedures but all other procedures (example echocardiography, colonoscopy) should only be taught if needed for that individual resident's future practice location/type. Opportunities should be given to attain this training after graduation if needed in the future.

As with many survey studies this study is limited by the response rate. We acknowledge that the response rate as estimated by the membership of CSIM (number of emails sent) is low; however we believe that we have captured opinions of a cross-section of practicing General Internists that can provide a basis for further study. As GIM has not previously been recognized as a distinct subspecialty by the Royal College of Physicians and Surgeons of Canada there is no master membership list of all practicing General Internists - the CSIM membership list is the best estimate at present. The intent was however to find a variety of general internists in a variety of practice locations as opposed to surveying the entire membership of CSIM. This goal was achieved with a wide variety of practice locations and types represented in the respondent sample. This is an opinion study and may not reflect what individual General Internists actually do in practice. Due to the nature of the CSIM listing, a minority of respondents were residents in a 4^th ^or 5^th ^year GIM training program. The survey was long and not everyone answered all questions.

## Conclusions

In conclusion, we believe that although a General Internist is a "generalist" there are also areas of distinct knowledge within GIM that must be mastered. We suggest that "generalism", at least in the context of GIM, does not mean a bit of knowledge about everything but instead mastery of core areas such as perioperative care, risk reduction, and management of common, emergent, and multiple internal medicine problems.

## Competing interests

The authors declare that they have no competing interests.

## Authors' contributions

SEC, APJ and RCO contributed to conception and design, analysis and interpretation of the data. RCO was primarily responsible for acquisition of data. SEC drafted the article; all three authors revised it critically for important intellectual content. All three authors gave final approval of the version to be published.

## Funding

RCO was the recipient of a Dean's Summer Student Research Project. The funding body had no role in study design, collection of data, writing of the manuscript or in decision to submit the manuscript for publication.
